# Donor-derived vancomycin-resistant enterococci transmission and bloodstream infection after intestinal transplantation

**DOI:** 10.1186/s13756-020-00845-z

**Published:** 2020-11-07

**Authors:** Carlos L. Correa-Martínez, Felix Becker, Vera Schwierzeck, Alexander Mellmann, Jens G. Brockmann, Stefanie Kampmeier

**Affiliations:** 1 Institute of Hygiene, University Hospital Münster, Robert-Koch-Straße 41, 48149 Münster, Germany; 2 Department of General, Visceral and Transplant Surgery, University Hospital Münster, Waldeyerstraße 1, 48149 Münster, Germany; 3 Institute of Medical Microbiology, University Hospital Münster, Domgakstraße 10, 48149 Münster, Germany

**Keywords:** Multidrug-resistant organisms, Vancomycin-resistant enterococci, Transplantation, Donor-derived infection, Whole genome sequencing, Bloodstream infection

## Abstract

**Background:**

Transplant recipients are at high risk for infections. However, donor-recipient transmission of multidrug-resistant organisms (MDROs) remains mostly unaddressed in the protocols of pre-transplant infection and colonization screening. Vancomycin-resistant enterococci (VRE) are MDROs that colonize the gastrointestinal tract and are associated with a significant burden of disease. Besides the high mortality of invasive VRE infections, chronic colonization leads to costly isolation measures in the hospital setting. Whereas most post-transplantation VRE infections are endogenous and thus preceded by colonization of the recipient, conclusive evidence of VRE transmission via allograft in the context of intestinal transplantation is lacking.

**Case presentation:**

We describe a donor-derived VRE infection after intestinal transplantation including small bowel and right hemicolon. The recipient, a 54-year old male with history of mesenteric ischemia and small bowel perforation due to generalized atherosclerosis and chronic stenosis of the celiac trunk and the superior mesenteric artery, developed an intra-abdominal infection and bloodstream infection after transplantation. VRE isolates recovered from the patient as well as from the allograft prior to transplantation were analyzed via whole genome sequencing. Isolates showed to be genetically identical, thus confirming the transmission from donor to recipient.

**Conclusions:**

This case underlines the relevance of donor-recipient VRE transmission and invasive infection in the context of intestinal transplantation, highlighting the need for preoperative MDRO screening that facilitates the prompt and effective treatment of possible infections as well as the timely establishment of contact precautions to prevent further spread.

## Background

Since the first successful small bowel transplantation in 1988 [1], intestinal transplantation has gradually become a reliable therapeutic option for patients with intestinal failure [2]. In Germany, an average of three intestinal transplantations were performed yearly between 2015 and 2019 [3]. Currently, this procedure is either conducted using isolated intestinal grafts (including the right hemicolon), as composite graft in combination with the liver or in combination with the stomach and pancreaticoduodenal complex as multivisceral graft [4, 5].

Infectious complications are common after intestinal and multivisceral transplantation, representing a relevant cause of morbidity and mortality among recipients, with reports indicating a risk of up to 90% for bacterial infections [6], 15–30% for cytomegalovirus infections and 30–50% for mycoses, respectively [7]. Of all bacterial infections observed in the early postoperative period, 60% evolve into bloodstream infections [8]. Solid organ transplant recipients are at high risk for infections caused by multidrug-resistant organisms (MDROs) [9] associated with a high attack rate and mortality, with studies reporting rates of up to 52% and 41%, respectively [10]. MDROs pose a serious threat to recipients. Due to their resistance to first- and second-line antibiotics as well as their spreading potential in the hospital setting [11], vancomycin-resistant enterococci (VRE) constitute globally relevant MDROs, having been defined as microorganisms of high priority for the development of new antimicrobials by the World Health Organization [12]. In Germany, the proportion of VRE among invasive *Enterococcus faecium* (*E. faecium*) isolates was 23.8% in 2018, above the European mean of 17.3% [13]. In the same period, this proportion amounted to 13% at our hospital.

The translocation of microorganisms from the lumen of an intestinal allograft to sterile sites in the recipient is a plausible pathophysiological mechanism of postoperative infection [8]. However, according to current definitions adopted in Europe and the United States, donor-derived infections are considered proven or certain when clear laboratory evidence of the presence of the same infectious agent in the donor and the recipient is available. Cases in which this evidence is only suggestive or strong but not conclusive, donor-derived infections are classified as possible or probable, respectively [14, 15]. Frequently, the lack of conclusive data hampers the confirmation of proven infections of donor origin, thus leading to underreporting [16].

In spite of the ubiquitous presence of enterococci in the intestinal flora, the role of VRE in donor-derived infections following intestinal and transplantation remains poorly addressed [17]. We present the case of a proven donor-derived VRE transmission and subsequent infection after intestinal transplantation confirmed by WGS.

## Case presentation

We report the case of a 54-year old male with intestinal failure due to a history of mesenteric ischemia and small bowel perforation caused by generalized atherosclerosis and chronic stenosis of the celiac trunk and the superior mesenteric artery. Initial surgical treatment consisted of a partial small bowel resection and creation of an end-jejunostomy, with subsequent total parenteral nutrition. Three years later, the patient was admitted at our center for intestinal transplantation. Since the remaining foregut organs (stomach, liver, pancreas and spleen) were only perfused by the inferior mesenteric artery via the anastomosis of Riolan, 2 weeks prior to transplantation (day-14) mesenteric revascularization was performed by means of an aorta-common hepatic artery bypass. Anal swabs taken before (day-14) and upon admission (day 0) were negative for vancomycin-resistant enterococci (VRE) and other MDRO. Prior to transplantation, a sample of allograft perfusion fluid was collected as part of the routinely pre-transplant evaluation and sent in for general microbiological testing (A, Table 1). Further pre-transplant donor screening included testing for hepatitis B virus, hepatitis C virus, human immunodeficiency virus, cytomegalovirus, syphilis and toxoplasmosis.

**Table 1:** Analyzed VRE strains, sample collection time and genetic relatedness

Code	Collection	Specimen	Genetic relatedness
A	Day 0	Allograft perfusion fluid	Cluster of identical isolates, 0 alleles of difference. 1 allele of difference to P6
P1	Day +2	Blood culture, peripheral	
P2	Day +2	Blood culture, central venous line	
P3	Day +2	Swab, intra-abdominal	
P4	Day +3	Swab, intra-abdominal	
P5	Day +3	Swab, intra-abdominal	
P7	Day +3	Swab, gallbladder	
P8	Day +3	Swab, duodenum	
P9	Day +3	Swab, stomach	
P6	Day +3	Swab, subcutaneus tissue	1 allele of difference to cluster
R	–	Reference genome, *Enterococcus faecium* Aus0004 [18]	288 alleles of difference to cluster

On day 0, small bowel transplantation and partial ascending colon transplantation were performed. Induction therapy consisted of two subcutaneous doses of alemtuzumab (30 mg) before and 24 h after transplantation. Postoperative antimicrobial therapy with piperacillin/tazobactam (4.5 g QID) and micafungin (100 mg OD) and an immunosuppressive regime with tacrolimus (1 mg BID) and steroids were started. After the patient developed fever and blood infection parameters increased on day +2, a surgical revision was performed, intra-abdominal swabs were taken for microbiological testing and blood cultures (peripheral and central venous line) were drawn (P1, P2 and P3, Table 1). Antibiotic therapy with vancomycin (1 g BID) was started on day +3 after *E. faecium* was detected in samples P1–P3. Further swabs (P4–P9) collected during a subsequent surgical revision were additionally sent in for microbiological testing. On day +4 antibiotic susceptibility testing results confirmed vancomycin resistance in the *E. faecium* isolates grown in samples P1–P3. Antibiotic therapy was modified from vancomycin to linezolid (600 mg BID), the central venous line was replaced as a preventive measure and contact precautions according to the hospital’s internal infection control standards were implemented. Microbiological results obtained on day +5 confirmed the presence of VRE in samples P4–P9 as well as in the allograft perfusion fluid (sample A).

**Fig. 1:**
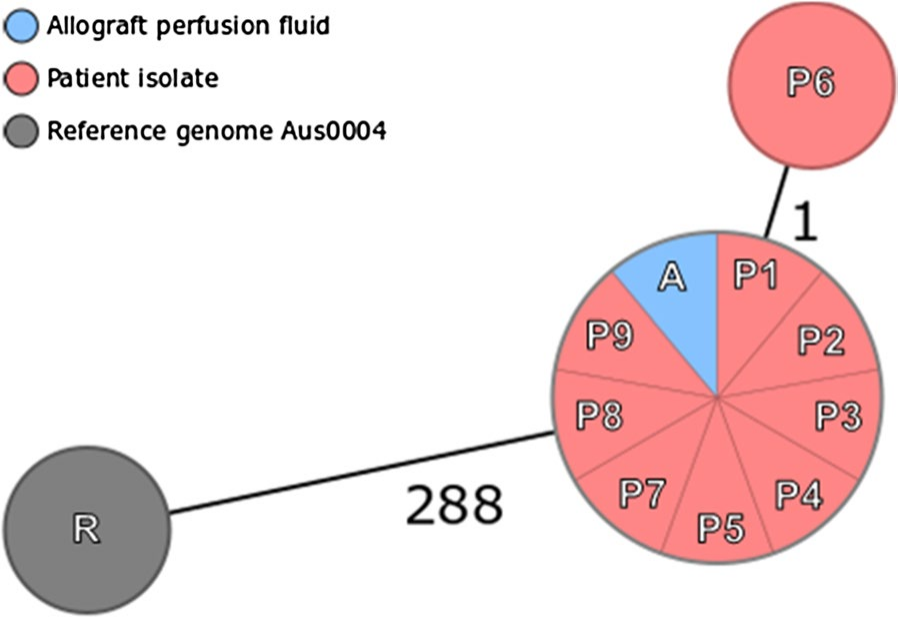
*Minimum spanning tree* illustrating the genetic relatedness of the analyzed VRE strains derived from the recipient (red), the allograft perfusion fluid (blue) compared to the reference genome *E. faecium* AUS0004, based on 1423 target genes. Every circle represents one genotype, connecting lines between circles indicate number of allelic differences. Close genetic relation is assumed if allelic difference is 3 or less

A possible VRE transmission from the donor to the patient suggested by the presence of VRE in the allograft perfusion fluid was further investigated by analyzing VRE strains isolated from recipient and allograft via WGS. For this purpose, the Illumina MiSeq platform (Illumina Inc., San Diego, CA, USA) was employed as previously described [19]. After quality trimming and de novo assembly of the sequences, the SeqSphere + software version 7.0.1 (Ridom GmbH, Münster, Germany) was used to compare coding regions gene by gene, performing a core genome multilocus sequence typing (cgMLST) and depicting the genetic relatedness of the strains in a minimum spanning tree. Sequences differing in three or less alleles were considered identical or very closely related [20]. As observed in Fig. 1, patient isolates P1 through P5 and P7 through P9 were identical to the allograft isolate A, grouped in a single genetic cluster from which isolate P6 differs in only one allele. All analyzed isolates belonged to the sequence type ST80 and carried the glycopeptide resistance determinant *vanB*. The difference of 288 genes between the cluster and the genetically non-related reference genome of the *E. faecium* Aus0004 (accession number: NC_017022) highlights the very close genetic relatedness of the isolates within the detected cluster.

After continuous therapy with linezolid, control blood cultures remained negative and intra-abdominal swabs taken upon relaparotomy on day +18 showed no VRE growth.

## Discussion and conclusions

Infections caused by VRE are known to have a significantly higher mortality than those caused by vancomycin-sensitive enterococci [21, 22], with immunocompromised and multimorbid patients being particularly susceptible [23, 24]. For this reason, pre-transplant screening for VRE is routinely performed on potential recipients at our hospital, seeking to detect a possible intestinal colonization that could give rise to an endogenous infection in the course of hospitalization. In the present case, a previous VRE colonization was ruled out, as indicated by two negative anal swabs taken upon admission. Therefore, transmission during the hospitalization period was initially considered the most probable origin of the postoperative VRE infection. The results of the WGS analysis performed on VRE isolates obtained from the patient and the allograft allowed to conclusively confirm a donor-derived transmission, defining it as proven/certain in accordance with current international concepts [14, 15].

VRE infections following solid organ transplantation display a mortality of 9–48%, reaching 56–80% in the first year after surgery [9]. A history of previous colonization has been shown to be strongly associated with the subsequent development of VRE infection. Colonization is common in solid transplantation patients, with up to 12% being positive for VRE in the pre-transplant period [25]. However, donor-derived VRE infections in previously non-colonized recipients is still an unaddressed issue [17]. Evidence is scarce, with only one report of VRE donor-recipient transmission following liver transplantation confirmed by genomic approaches in the United States [26]. Currently, no reports yielding information on VRE transmission after intestinal transplantation are available.

Considering the overall rising incidence of VRE infections worldwide [27], increasing donor-derived VRE colonization and infection cases are to be expected in the next years. While VRE infections are correctly diagnosed and reported in most cases, colonization cases may be frequently overlooked when VRE screening procedures are not routinely performed in the context of solid organ transplantation, contributing to an unnoticed spread of these microorganisms. In this case, microbiological pre-transplant allograft screening was performed as part of a routine microbiological analysis aiming at detecting a broad range of bacterial microorganisms. Employing selective culture media might have enhanced and accelerated VRE isolation and identification in the allograft perfusion fluid. Thus, the inclusion of specific microbiological testing for VRE in the protocols for pre-transplant allograft screening should be taken into consideration, as well as further targeted MDRO screening techniques. This constitutes a feasible strategy for preoperative risk assessment that would enable the immediate administration of VRE-specific antibiotic therapy upon first clinical signs of infection in the recipient. Hence, time to administration of effective therapy could be shortened by 1–2 days, potentially leading to better clinical outcomes and ultimately contributing to the overall reduction of post-transplant morbidity and mortality. Additionally, VRE testing of recipients should be carried out by means of anal swabs in patients with history of previous colonization or risk factors for becoming colonized. In conclusion, this case described a donor-derived VRE transmission after intestinal transplantation, highlighting the relevance of these pathogens in the development of serious postoperative infections as well as the need to consider VRE in pre-transplant screening protocols.

## Data Availability

The datasets generated and/or analysed during the current study are available in the ENA repository (PRJEB40484).

## Abbreviations

BID: *Bis in die*, twice daily
cgMLST: Core genome multilocus sequence typing
CT: Computed tomography
*Enterococcus faecium*: *E. faecium*
MDRO: Multidrug-resistant organism
MDRGN: Multidrug-resistant Gram-negative bacteria
OD: *Omne in die*, once daily.
QID: *Quarter in die*, four times daily.
VRE: Vancomycin-resistant enterococci
WGS: Whole genome sequencing
